# *TGFBI* mutation screening and genotype-phenotype correlation in north Indian patients with corneal dystrophies

**Published:** 2010-07-29

**Authors:** Preeti Paliwal, Arundhati Sharma, Radhika Tandon, Namrata Sharma, Jeewan S. Titiyal, Seema Sen, Punit Kaur, Divya Dube, Rasik B. Vajpayee

**Affiliations:** 1 Laboratory of Cyto-Molecular Genetics, Department of Anatomy, All India Institute of Medical Sciences, New Delhi, India; 2 Dr. Rajendra Prasad Centre for Ophthalmic Sciences, All India Institute of Medical Sciences, New Delhi, India; 3 Department of Biophysics, All India Institute of Medical Sciences, New Delhi, India; 4 Centre for Eye Research Australia, University of Melbourne, Australia

## Abstract

**Purpose:**

To screen a cohort of corneal dystrophy patients from North India for mutations in the transforming growth factor beta induced (*TGFBI*) gene, to correlate genotypes to phenotypes, to describe structural implications of various mutations on protein function, and to discuss the implications for diagnosis.

**Methods:**

Eighty affected individuals from 61 unrelated families, who were diagnosed with autosomal dominant granular and/or lattice corneal dystrophy, were recruited for the study. Detailed clinical evaluation was undertaken for these patients to establish their corneal phenotypes. Genomic DNA was isolated from peripheral blood samples and all exons of *TGFBI* were screened for mutations by polymerase chain reaction (PCR) and direct DNA sequencing. Protein molecular dynamics (MD) simulations were performed for the mutations detected to assess the changes in protein structure.

**Results:**

The most common mutations seen were Arg555Trp and Arg124Cys. Two novel mutations, Ser516Arg (c.DNA1548C>G), with a phenotype similar to granular corneal dystrophy I (GCDI), and Leu559Val (c.DNA1675T>G), with an atypical phenotype closely resembling epithelial basement membrane dystrophy/map dot fingerprint dystrophy, were identified. Protein modeling studies involving wild type and mutant protein indicated that the Leu559Val is a destabilizing mutation and that Ser516Arg could adversely affect the specific binding of Fas1 domain 4 with other proteins. In addition, two single-nucleotide polymorphisms, rs4669 and rs11331170, were also identified. Mutations were not identified in 8 affected individuals, 6 of whom were diagnosed with bowman layer dystrophy and 2 with lattice corneal dystrophy.

**Conclusions:**

This is the first comprehensive report of *TGFBI* mutations covering a large part of North India. Identification of novel mutations, the presence of phenotypic variability, and the genetic heterogeneity seen in our cases stress the need for mandatory screening of *TGFBI* for precise diagnosis and classification of corneal dystrophies.

## Introduction

Corneal dystrophies (CDs) are defined as a group of inherited, bilateral, symmetric, slowly progressive corneal diseases without any relationship to environmental or systemic factors. Recently, the International Committee for Classification of Corneal dystrophies (IC3D) has provided a new classification that integrates phenotypic description, histopathological examination, and genetic analysis [1]. Since the initial discovery by Munier et al. [2] of mutations in the transforming growth factor beta induced gene (*TGFBI*; also known as BIGH3), located on chromosome 5q31 that cause autosomal dominant CDs, more than 38 mutations have been reported in association with different phenotypic variants [1,3-5]. Specific mutations in *TGFBI* are associated with distinct forms of CDs although both interfamilial and intrafamilial variability have been described in patients belonging to different ethnic backgrounds [6].

*TGFBI* codes for an extracellular matrix protein, keratoepithelin. The protein has 683 amino acids and four internal repeat domains, with structural homology to fasciclin-1 of Drosophila [7]. The gene is expressed in many tissues, including corneal epithelium, and its mutations in this tissue lead to accumulation of abnormal keratoepithelin protein [8]. Keratoepithelin was initially discovered as a 69-kDa secretory protein in a human lung adenocarcinoma cell line [9]. The protein includes an NH_2_-terminal signal peptide that is excised during export to the extracellular matrix, four fasciclin-like domains, and a COOH-terminal Arg-Gly-Asp (RGD) sequence, which is a putative integrin-binding motif.

Keratoepithelin has been suggested to bind to integrins, which are cell surface receptors that trigger downstream signaling [10-12], as well as to different types of collagens [13,14].

Four distinct autosomal dominant corneal dystrophies are associated with mutations in *TGFBI;* these include granular CD type I, granular CD type II (Avellino), granular CD type III (Reis- Bücklers dystrophy), and Lattice CD (LCD). Of these, Groenouw granular CD I (CDGG1, OMIM 121900) is the most common type of autosomal dominant corneal dystrophy and is characterized by the presence of multiple discrete crumb-like, gray-white corneal opacities [15]. Histopathologically, these opacities stain red with Masson trichrome [16]. Typical granular corneal dystrophy, i.e., GCDI is caused by an Arg555Trp mutation [2]. The LCD (OMIM 122200) form is characterized by fine, refractile lines that form a branching network [17]. On histopathology, the deposits in LCD stain positively with Congo red and are birefringent under polarized light. Various subtypes (I, IIIA, I/IIIA, IIIB, and IV) of LCD occur, with classification based on the underlying mutations in the *TGFBI*.

The present study, conducted on eighty individuals, was aimed at identification of the underlying mutations in the autosomal dominant granular or lattice CDs. We report, for the first time, the mutation spectrum of patients encompassing the population in a major part of North India.

## Methods

The study protocol adhered to the tenets of Declaration of Helsinki and was approved by the Ethics Committee of the Institute (All India Institute of Medical Sciences, AIIMS). The patients presented at the Cornea Clinic of the Dr. Rajendra Prasad Center for Ophthalmic Sciences, AIIMS, New Delhi, India). All CDs were diagnosed by clinical evaluations and by histopathological evidence wherever possible. Clinical examination included routine slit lamp biomicroscopy, confocal microscopy, and specular microscopy. All patients who were bilaterally affected and who did not have any other systemic involvement formed the study group.

A total of 80 patients from 61unrelated families were recruited after obtaining their informed consent. Of these 80 patients, 51 (64%) were clinically diagnosed with granular CD, 23 (29%) were diagnosed with lattice CD, whereas in six (7.0%) patients the subtype of CD could not be ascertained based on the clinical features. Detailed family history was collected and pedigree charts were constructed for all patients.

The control population consisted of 100 unrelated and healthy individuals who had no history of any ocular disease in their family. From both groups – the controls and patients, along with their available family members – 5 ml samples of peripheral blood were collected in EDTA after obtaining informed consent.

Histopathological data were available for correlation with clinical diagnosis in seventeen patients who underwent surgical intervention (keratoplasty). For histopathology, the corneal sections were processed by standard methods involving sectioning of the tissue samples; sections were then stained with Congo red and/or Masson trichrome and analyzed by light microscopy for the presence of amyloid and/or hyaline deposits. The presence of amyloid was confirmed by birefringence when viewed under polarized light.

The peripheral blood samples were processed for Genomic DNA extraction using DNAeasy kits (Qiagen, GmBH, Hilden, Germany). These samples were used for amplification of all 17 exons of *TGFBI* using primers and conditions as described previously  [2]. Briefly, the cycling conditions were 5 min initial denaturation at 95 °C, 30 cycles of 94 °C for 1 min, 58 °C/55°C for 1 min, 72 °C for 1 min, and a final extension at 72 °C for 5 min. The reaction mixture of 25 μl was prepared using 200 ng genomic DNA, primers (0.5 µM each), MgCl_2_ (1.5 mM), deoxyribonucleotide triphosphates (dNTPs; 0.2 mM), 1× PCR buffer (containing 10 mM TRIS-HCl, pH 8.3, 50 mM KCl; Roche, Applied Biosystems, Foster City, CA), and Taq polymerase (0.5 U; Roche, Applied Biosystems). Primer sequences used for amplification are listed in Table 1. The amplified PCR products were subjected to gel purification using QIAmp gel extraction kits (Qiagen, GmBH) and the purified PCR products were screened for sequence changes by bidirectional sequencing. Sequencing was performed using BigDye Terminator Mix, version 3.1 (Applied Biosystems, Inc. [ABI], Foster City, CA) and were then analyzed on an ABI-3100 Genetic Analyzer (ABI). Nucleotide sequences were compared with the published sequence of *TGFBI* (GenBank NM_000358) for analysis of sequence changes.

**Table 1:** Table showing the *TGFBI* primer sequences.

**Sample number**	**Exon**	**Forward primer **	**Reverse primer **	**Product size (base pairs)**	**Annealing temp (°C)**
1	1	5’-CCGCTCGCAGCTTACTTAAC-3’	5’-AGCGCTCCATGCTGCAAGGT-3’	360	58
2	2	5’-GTGGACGTGCTGATCATCTT-3’	5’-TCCTGGCTGGTTACAGATAC-3’	170	55
3	3	5’-GAGAATGCCATGTCCTTGTG-3’	5’-GCTGTGGAGGCAACTTAGTG-3’	280	58
4	4	5’-CCCCAGAGGCCATCCCTCCT-3’	5’-CCGGGCAGAAGGAGGTCATC-3’	225	55
5	5	5’-TAAACACAGAGTCTGCAGCC-3’	5’-TTCATTATGCACCAAGGGCC-3’	260	55
6	6	5’-TGTGTTGACTGCTCATTCTT-3’	5’-CATTCAGGGGAACCTGCTCT-3’	316	55
7	7	5’-AAGTGTGCCAAGTTGACCTC-3’	5’-GGCAGGTGGTATGTTCATCT-3’	588	55
8	8	5’-AGAAGGCGAGGAGGATCTGG-3’	5’-CAGTGGCCGAGAAGCTGTGA-3’	508	55
9	9	5’-CATTCCTGCTGATGTGTGTCATGC-3’	5’-GGGTGCTGTAAATCGGAGAGTGTT-3’	315	55
10	10	5’-TCTGGACCTAACCATCACCC-3’	5’-CAGGAGCATGATTAGGACC-3’	206	55
11	11	5’-CTCGTGGGAGTATAACCAGT-3’	5’-TGGGCAGAAGCTCCACCCGG-3’	223	55
12	12	5’-CATTCCAGTGGCCTGGACTCTACTATC-3’	5’-GGGGCCCTGAGGGATCACTACTT-3’	318	55
13	13	5’-CCTCCTTGACCAGGCTAATTA-3’	5’-GGCTGCACTGAAGGTTGTG-3’	300	55
14	14	5’-CTGTTCAGTAAAACTTGCT-3’	5’-CTCTCCACCAACTGCCACAT-3’	260	58
15	15	5’-ACAGCATCTCACCTCAGTGT-3’	5’-AACCTAGCAGGCATCTTACC-3’	360	55
16	16	5’-GCTTGCACAACTTATGTCTG-3’	5’-CAGGTCTGCAATGACTTC-3’	251	55
17	17	5’-CCTGTCCTTGAGATTCTGA-3’	5’-GAGGCTGGATTGCTTGATTC	489	55

The crystal structure of the Fas1 domain 4 (amino acid residues 502–633) of the transforming growth factor-beta induced protein, Big-H3, available in a public database (PDB Id: 2VXP), was used as the starting point for all the molecular modeling studies. Mutants (Ser516Arg and Leu559Val) were generated by altering the corresponding residues and the resulting native and mutated structures were minimized separately by employing molecular dynamics simulation protocols using the program Discovery Studio 2.0 (Messrs Accelrys, San Diego, CA).

### Molecular dynamics (MD) simulation

The effect of the mutation on the overall conformation of the Fas1 domain 4 of *TGFBI* was checked by extensive MD simulations performed on a fully hydrated model of both the wild type and mutant structures, using an explicit spherical boundary with harmonic restraint with a Radius of Sphere as 25.0 Å (angstrom). The Standard Dynamics Cascade Protocol was used with a time step of one femtosecond (fs). The first step in the MD simulation was the energy minimization of the hydrated model. The minimized hydrated complex was then subjected to a MD simulation in three stages. In the first stage, the temperature of the system was raised from 50 to 300 K over 2 picoseconds (ps), followed by equilibration for 20 ps, and the final production run was performed for 0.5 ns (ns). The conformations obtained after every 0.1 ns were saved and analyzed for the variations induced in the protein conformation due to the mutations. To analyze the probable quaternary structures (assemblies) formed by this protein, both the mutant and the native MD conformers were submitted to the PISA server.

## Results

**Figure 1:**
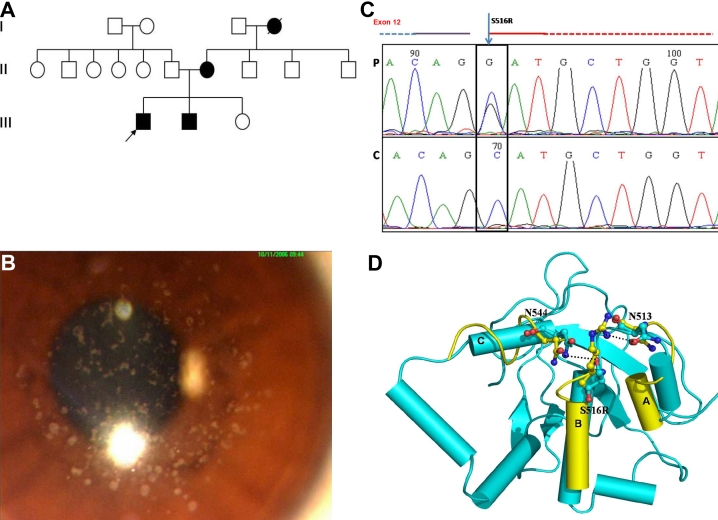
Family 1, showing the S516R mutation in the transforming growth factor beta Induced (*TGFBI*) gene. **A**: Pedigree of the family. Filled boxes represent affected individuals. Open boxes represent unaffected individuals. Arrowhead indicates the proband. Filled circle with a slash indicates a deceased individual. **B**: Slit lamp photomicrograph of the affected individual. The representative clinical photograph shows the presence of discrete gray-white deposits in the right eye of the proband, with clear intervening stroma resembling granular corneal dystrophy. **C**: Partial nucleotide sequence of the transforming growth factor beta induced (*TGFBI*) gene. The chromatogram of the patient (P) is shown compared to a control (C). A heterozygous C>G substitution, marked by the arrowhead, is shown. The black box denotes the nucleotide that causes the missense mutation resulting in amino acid substitution of the Serine (S) at amino acid position 516 with Arginine (R). The partially dashed blue line at the top left of the chromatogram marks the intronic region, while the red line on the right marks the start of the exon. **D**: Protein modeling in S516R mutation showing the superimposition of S516R mutant (yellow) and wild type conformers (cyan). Only the mutant structures, where the deviations were observed, are shown in the figure. The conformational changes in the secondary structure elements are shown for amino acid residues 505–511 (A), 516–525 (B) and 544–550 (C). The changes observed in molecular interactions (Hydrogen bonds) are also marked by dashed lines.

**Figure 2:**
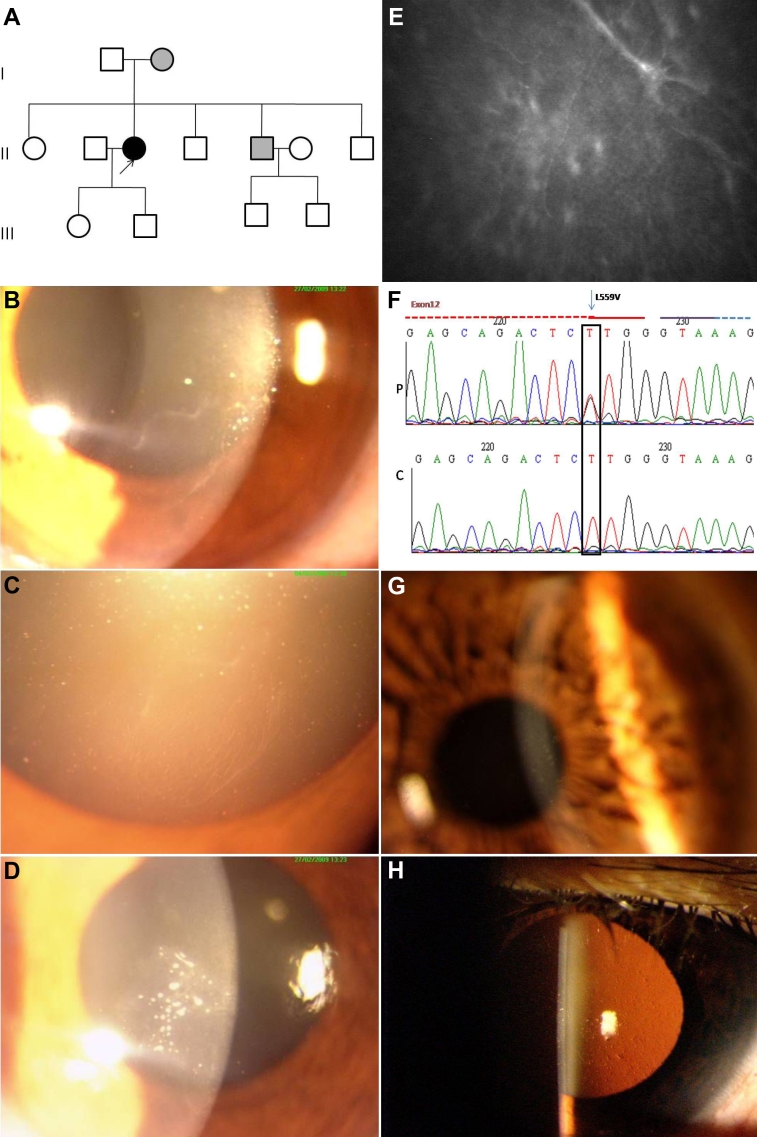
Family 2 showing the L559V mutation in the transforming growth factor beta induced (*TGFBI*) gene. **A**: Pedigree of the family. Filled boxes represent affected individuals. Open boxes represent unaffected individuals. Arrowhead indicates the proband. Gray boxes mark the affected individuals with a variable phenotype. **B**-**E**: Slit lamp and in-vivo confocal photomicrographs of the affected individual. The representative clinical photographs of the proband show the presence of a map-like structure in the right eye (**B**). Retro illumination photomicrograph demonstrates a fingerprint-like pattern in the right eye (C). The left eye of the proband shows multiple dot-like opacities (D). The map pattern in the sub-epithelial region, which was confirmed by in vivo confocal microscopy, is shown (2E). **F**: Partial nucleotide sequence of the transforming growth factor beta induced (*TGFBI*) gene. The chromatogram of the patient (P) is shown compared to a control (C). A heterozygous T>G substitution, marked by the arrowhead, is shown. The block denotes the nucleotide with missense mutation that results in amino acid substitution of Leucine at 559 position with Valine. The partially dashed blue line on the top right of the chromatogram marks the start of intron while the red line marks the exonic region. **G**-**H**: Representative slit lamp photomicrographs of the affected family member. Representative clinical photographs of the affected family member (**G**) showing very fine dot like opacities in the right eye. The indirect slit lamp retroillumination image (**H**) shows the presence of multiple dimple-like structures in the affected cornea.

Of the total patients studied, 55% were females and 45% were males. Most patients reported CD with an age at onset in their second decade. Consanguinity was not seen in any of the families. Seven patients with lattice CD and 15 with granular CD had a positive family history of the disease. Mutations Arg124Cys and Arg555Trp were the ones most commonly seen in most the patients. The Arg555Trp mutation was seen in 35 patients (44%) with GCD I and 19 patients (24%) showed typical lattice CD (LCDI) with an underlying Arg124Cys mutation. The other changes observed included two novel mutations, Ser516Arg (c.DNA1548C>G) and Leu559Val (c.DNA1675T>G), in patients clinically characterized to have granular CD and a variant of granular CD/epithelial basement membrane dystrophy, respectively. Pedigrees with segregation of the novel mutations in the available family members are shown in Figure 1 and Figure 2.

Mutations Thr538Pro and Arg124His have been identified for the first time in a population from India. Other reported mutations identified include Arg124Leu, His626Arg, and Ala546Asp and the phenotypes of the patients conformed to those reported earlier (Table 2). In addition, we also identified two single nucleotide polymorphisms, rs4669 and rs11331170, in the *TGFBI* gene. All of these mutations were present in the heterozygous state in the probands and were absent in 100 unrelated unaffected individuals of the control group, based on direct sequencing.

**Table 2:** Transforming growth factor beta induced (*TGFBI*) mutations seen in the present study. The table shows the identified mutations and the associated phenotypic features seen in our patients. These mutations have been previously reported and documented [1]. F- Female; M- Male; FCCF-Finger Counting Close to Face; FC1/2m-Finger Counting from half a meter; FC1/4m-Finger Counting from a quarter of a meter; MT- Masson Trichrome; CR-Congo Red; NR- Not Recorded, PL-Perception of Light. R>L*- right eye affected to a greater extent than the left eye, p-partial, P- present, A- absent.

**Sample number**	**Family Number**	**Mutation Segregation**	**Phenotype**	**Age/sex**	**Age at onset**	**Corneal erosion**	**Visual acuity**	**Corneal pachymetry**		**Histology**		**Mutation detected**	**Exon of *TGFBI* affected**	**cDNA position**
1	16.1	Son	Stromal opacities up to half to two-thirds depth, Focal and refractile lines.	18/M	15	P	6/9	6/9	571	595	-	A546D	12	c.1637C>A
2	16.2	Mother	Polymorphic opacities combined with lattice like lines extending to periphery and central cornea	43/F	36	P	6/9p	6/12p	521	463	-	A546D	12	c.1637C>A
3	39.1	Sporadic case	Multiple opacities coalescing into one, affecting Bowmans layer and anterior stroma	70/M	40	P	6/36	PL-ve	549	540	MT +ve	R124L	4	c.371G> T
4	20.1	Sporadic case	Thick, ropy lattice lines in the anterior stroma.	24/M	19	P	FC1/2m	5/60	566	403	CR +ve	T538P	12	c.1612A>C
5	69.1	Father	discrete disc and ribbon like opacities in the anterior stroma	50/M	30	A	6/9	6/9	-	-	-	R124H	4	c.367G>C
6	69.2	Daughter	Discrete ring- and crumb-shaped opacities distributed in a radiating pattern in the anterior stroma of both eyes	19/F	15	A	6/6	6/6	-	-	-	R124H	4	c.367G>C
7	155.1	Mother	Discrete rings, crumbs, and a few ray-like, linear, gray-white opacities in the anterior axial corneal stroma	53/F	40	P	6/9	6/9	494	484	-	R124H	4	c.367G>C
8	155.2	Daughter	Discrete rings, crumbs, and a few raylike, linear, gray-white opacities in the anterior axial corneal stroma R>L	32/F	22	A	6/12	6/9	521	519	-	R124H	4	c.367G> C
9	155.3	Son	Unilateral presentation with a single discrete opacity in the right eye	28/M	NR	A	6/6	6/6	495	490	-	R124H	4	c.367G> C
10	134.1	Son	Thick lattice lines in deep stroma, L>R	52/M	50	P	6/6	6/18	561	565	-	H626R	14	c.1877A>G
11	134.2	Mother	Thick lattice lines extending toward the periphery R>L	78/F	59	P	FCCF	FC1/4m	553	558	-	H626R	14	c.1877A>G

Of the eighty cases, *TGFBI* mutations were not identified in 8 patients. Of these 8 cases, 6 were clinically diagnosed to have bowman layer dystrophy and 2 had lattice CD.

### Clinical and histopathological features of patients with novel mutations

#### Family 1 - Mutation S5156R

#### Clinical details

The proband (Figure 1A), a 19 year old male, had multiple small discrete opacities in the anterior stroma with clear intervening stroma and limbus. (Figure 1B) The patient had no photophobia or corneal erosions. His best corrected visual acuity was 6/12 in both eyes. He had suffered a diminution of vision since the age of 11 years. Corneal pachymetry revealed normal thickness (496 [right eye]; 490 [left eye]). Specular microscopy revealed a normal endothelial cell count in both eyes (2,886 [right eye], 2,941 [left eye]). His 17-year-old brother and his 46-year-old mother were also affected and had a similar phenotype. The deep stroma of all of the affected family members was clear and only the anterior stroma was affected. No surgical interventions were undertaken in view of the good visual acuity of the patient.

#### Mutation analysis

Direct sequencing analysis of *TGFBI* revealed an underlying change in exon 12 at codon 516 with the amino acid serine being replaced by arginine (Ser516Arg; Figure 1C). The mutation was present in the heterozygous state in the proband and segregated in all of the affected family members and in none of the unaffected members of the family. The previously reported SNP rs4669 was also identified in all the affected members of the family. This mutation was absent in 100 controls who were also screened for underlying changes by direct sequencing.

#### Protein modeling

A comparison of the MD conformers in the mutants Ser516Arg and wild type structures revealed a minor variation in the overall geometry of the protein, with a root mean square deviation (rmsd) variation of 0.7 Å for Ser516Arg in the backbone atoms. The stability of the protein was conserved even in the presence of the Ser516Arg mutation. Closer analysis revealed a subtle deviation in the secondary structure element, mainly in the regions housing the mutant and its neighboring interacting residues, which comprised the secondary sites at amino acid positions 505–511, 544–550 and 516–525 (Figure 1D).

The mutation of Ser516 alters the characteristic of the residue in terms of both the nature and the length of the side chain. The longer and basic arginine residue in the mutated *TGFBI* forms an additional hydrogen bond with the side chain of Asn513, whereas in the wild type, Ser516 formed bonds with the asparagine (Asn544) side chain, resulting in shortening of the helical conformation in the mutated protein. Due to the change in the interacting residues, a subtle change in the conformation of the loops involving residues 513 and 544 was also observed. The PISA server analysis shows that mutation results in an overall gain in solvation free energy of the protein molecule. The solvation free energy is the amount of energy released when two protein molecules interact and is an indicator of the possibility of interactions between two protein molecules. Thus, the increase in energy indicates that due to the mutation Ser516Arg, the interaction of the present protein with other proteins is affected.

#### Family 2 – Mutation L559V

#### Clinical details

Slit lamp examination of a 42-year-old female patient (Figure 2A) revealed map-like opacity in the inferonasal quadrant of the cornea (almost adjacent to the pupillary area) in the right eye (Figure 2B,C). The left eye of the patient had multiple dot-like white opacities in the inferonasal quadrant overlying the pupillary areas (Figure 2D). The presence of map-like opacities in the right eye was confirmed by in-vivo confocal microscopy (Figure 2E). The epithelium of both eyes had a beaten metal appearance. Her uncorrected visual acuity was 6/18 in both eyes. Genetic analysis identified a missense mutation whereby the amino acid leucine at position 559 was replaced with valine (Leu559Val; Figure 2F). Examination of all members of the family identified the mother and one brother as affected, although neither reported any visual complaints. The visual acuity of the brother, who was 40 years old at the time of examination, was 6/6 in both eyes. The mother, aged 68 years, had best corrected visual acuity of 6/12 in both eyes. Slit lamp examinations identified dot-like opacities in both the mother and the affected brother (Figure 2G,H). The same Leu559Val mutation was identified in the mother and the brother, while none of the unaffected family members or any of the 100 controls showed this change.

#### Protein modeling

**Figure 3:**
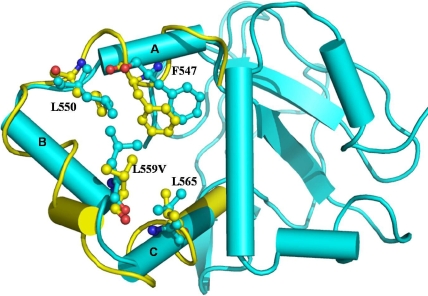
: Protein modeling studies showing the superimposition image of L559V mutant (yellow) and wild type conformers (cyan). Only the mutant structures in which deviations were observed are shown. The conformational changes in the secondary structure elements are marked for the amino acid residues 544–549 (A), 552–560 (B) and 563–572 (C), respectively. The changes were mainly observed in non-covalent hydrophobic interactions.

A comparison of the MD conformers in the mutants L559V and wild type structures revealed minor variation in the overall geometry of the protein with an rmsd variation of 0.9 Å for Leu559Val in the backbone atoms, which was checked with the ProsaII server [18]. The stability of the protein and odds of misfolding in the mutant structures was checked with the Eris server [19]. The overall geometry of the Leu559Val mutant conformers was conserved; however, the protein stability calculations suggested that this mutation had resulted in loss of protein stability. A minor difference in the secondary structure elements, mainly in the regions in and around the mutated residues, was also observed (Figure 3).

## Discussion

**Figure 4:**
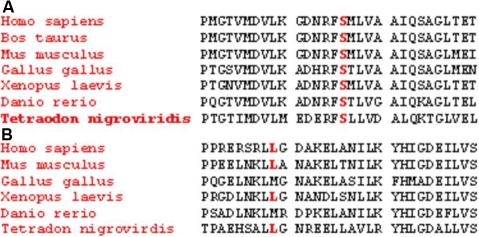
Multiple sequence alignment of the transforming growth factor beta induced (*TGFBI*) gene from different species. **A**: The amino acid Serine (S) at position 516, conserved in all of the orthologs, is highlighted in red. **B**: The amino acid Leucine (L) at position 559, highlighted in red, is conserved during the course of evolution over a large range of species.

Mutational analysis of the 80 patients from North India revealed typical missense mutations in exon 4 (Arg124Cys) and exon 12 (Arg555Trp) of *TGFBI* in most the patients with lattice corneal dystrophy (LCD) and granular corneal dystrophy (GCD), respectively. This work represents the first report of mutations Arg124His and Thr538Pro from India. Two novel mutations, Ser516Arg and Leu559Val, were identified in patients who were clinically diagnosed with GCD and an atypical variant of GCD, respectively. Both mutations occur in the highly conserved fourth fasciclin like domain of keratoepithelin. These positions are well conserved among the orthologs, like *Mus musculus, Xenopus laevis, Tetradon nigrovirdis,* etc., when evolutionary comparisons were made using the SIFT (Sorting Intolerant From Tolerant) tool (Figure 4A,B).

The phenotype of the two siblings with Ser516Arg resembled that of granular corneal dystrophy I (GCDI). These patients did not undergo any surgical intervention due to their good visual acuity. Hence, histopathology was not performed to confirm the nature of the deposits in these patients. In a recent report on Chinese families, two novel mutations at positions Arg514Pro and Phe515Leu were identified in *TGFBI* with phenotypes resembling LCD [20]. We identified a mutation in the adjacent amino acid with a phenotype resembling GCD. The two previously described mutations (Arg514Pro and Phe515Leu) occur in exon 11 of the *TGFBI* gene, while the mutation Ser516Arg seen in our Family 1 occurs in the first nucleotide of exon 12. This change in the first nucleotide of exon 12 might affect the pre-mRNA splicing, leading to either exon skipping or intron retention, which would consequently result in an altered protein structure. This may be a possible reason for the different phenotype seen in our family when compared to the changes at the adjacent codons that gave rise to the lattice-like features reported by Zhong et al. [20].

**Figure 5:**
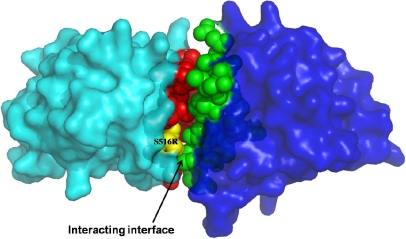
The quaternary association of S516R mutant (cyan) and wild type (dark blue) structures in surface representation, as obtained using the Pisa server. The mutation is colored yellow and the interacting hydrophobic groove in the mutant is colored red, while the respective interacting surface of the wild type structure is shown in green.

Earlier molecular modeling and mutational studies on the Fas1 domain 4 of the corneal protein TGFBI suggested that the mutation Ser516Arg is part of the predominant hydrophobic groove (F515-L531) and is present on the outer surface of the protein (Figure 5), which had a role in protein–protein interaction [8]. Protein modeling of Ser516Arg mutation revealed that the change decreases the hydrophobicity which is required for specific protein affinity. This adversely affects its association and specific binding interactions with that of other proteins or its longer side chain could possibly interfere with protein secretion.

The patient with the Leu559Val mutation had a map pattern with fingerprint-like structures in the sub-epithelial region, in addition to granular gray-white opacities. This phenotype did not conform to typical GCD or its subtypes, as seen in Figure 2. The affected mother and brother did not complain of any ocular problems, but harbored small dot-like opacities that did not affect their visual acuity. A highly variable phenotypic presentation was seen for the Leu559Val mutation in the affected members of Family 2. Mutation at the adjacent amino acid leucine (Leu558Pro) caused an atypical phenotype characterized as amyloid on histopathology [3]. None of the patients identified with the Leu559Val mutation in the present study required any surgical intervention; therefore, the nature of deposits could not be ascertained on histopathology. Molecular modeling studies on the Leu559Val mutant of *TGFBI* have shown that this mutant has minor changes in the secondary structure that affect the stability of the protein. The mutation Leu559Val occurs in the fourth domain of keratoepithelin; previous studies have shown that mutations in the fourth domain alter the structure of the protein, leading to its accumulation [8].

The cases harboring the novel mutations identified in the present study underline the fact that phenotypic presentation of the disease can be highly variable and can overlap. This also highlights the fact that a single nucleotide change leading to an amino acid substitution alters the structure of keratoepithelin, leading to a difference in the nature of its deposition and its accumulation pattern.

To the best of our knowledge, this is the first comprehensive report of CDs in a patient population encompassing a large part of North India. Such cases, with phenotypic variability among the autosomal dominant CDs, add to the difficulties in their classification, especially in cases where no histopathological evidence is available. With no underlying mutation seen in eight cases, our study also supports the presence of genetic heterogeneity of the disease, as reported earlier [21,22].

In conclusion, the study identified two novel mutations that add to the existing mutation spectrum of the *TGFBI* dystrophies. Our results reiterate the need for *TGFBI* screening as an essential requisite, along with histopathological evidence wherever possible, for precise diagnosis and classification of these dystrophies. This would aid in the proper treatment, management, and genetic counseling of CD patients.
